# New type of Al-based decagonal quasicrystal in Al_60_Cr_20_Fe_10_Si_10_ alloy

**DOI:** 10.1038/srep22337

**Published:** 2016-03-01

**Authors:** Zhanbing He, Haikun Ma, Hua Li, Xingzhong Li, Xiuliang Ma

**Affiliations:** 1 State Key Laboratory for Advanced Metals and Materials, University of Science and Technology Beijing, Beijing 100083, China; 2 Nebraska Center for Materials and Nanoscience, University of Nebraska, Lincoln, NE 68588-0656, USA; 3 Shenyang National Laboratory for Materials Science, Institute of Metal Research, Chinese Academy of Sciences, Wenhua Road 72, 110016 Shenyang, China

## Abstract

A new kind of decagonal quasicrystal (DQC) with a periodicity of 1.23 nm was observed in the as-cast quaternary Al_60_Cr_20_Fe_10_Si_10_ alloy. The intensity distribution of some spots in the selected-area electron diffraction pattern along the tenfold zone axis was found to be different from other Al-based DQCs. High-angle annular dark-field scanning transmission electron microscopy was adopted to reveal the structural features at an atomic level. Both the tenfold symmetry and symmetry-broken decagonal (D) clusters of 1.91 nm in diameter were found, but with structural characteristics different from the corresponding D clusters in the other Al-based DQCs. The neighboring D clusters are connected by sharing one edge rather than covering, suggesting the tiling model is better than the covering model for structural description.

Two-dimensional (2D) decagonal quasicrystals (DQCs) have been attracted widely interest^1^,^2^,^3^,^4^,^5^,^6^,^7^,^8^,^9^,^10^,^11^ since the discovery in the rapid solidified Al-Mn alloys by Bendersky^12^ and in Al-Fe alloys by Fung *et al.*^13^ The DQCs were also achieved by slow solidification in the binary Al-Co alloys^14^ and ternary Al-TMI-TMII systems (TMI, II: different transition metals)^4^. As one of crucial questions of the DQCs, the crystal structures of the ternary Al-Cu-Co^15^,^16^,^17^,^18^,^19^,^20^,^21^,^22^, Al-Ni-Co^23^,^24^,^25^,^26^,^27^,^28^,^29^,^30^,^31^,^32^,^33^,^34^,^35^,^36^,^37^, Al-Ni-Fe^38^,^39^, Al-Ni-Ru^40^, and Al-Mn-Pd^41^,^42^,^43^,^44^,^45^,^46^ DQCs have been extensively studied by both X-ray diffraction and transmission electron microscopy (TEM), and the structural details of these DQCs have been revealed at an atomic scale. Although DQCs have also been found in the quaternary alloys, e.g. in the Au-Cu-Co-Si^47^,^48^,^49^, Al-Ni-Co-Tb^50^, Ga-Fe-V-Si^51^, Al-Co-Cu-Ni^52^, Al-Cr-Fe-Cu^53^, and Al-Mn-Fe-Ga^54^ systems, the structural information at an atomic scale for the quaternary DQCs is far less known than those in the ternary Al-Cu-Co, and Al-Ni-Co DQCs.

TEM has been widely adopted to reveal the local structures of DQCs^8^. Especially, the high-angle annular dark-field (HAADF) scanning transmission electron microscopy (STEM) shows advantages over other methods because it presents not only the structural information down to the scale of picometer, but also reveals the local chemical information simultaneously^55^. For example, the positions of the mixed sites of Al and TM (MSs), as well as the TM atoms in Al-Co-Ni DQC were determined directly from the different intensities of image spots in the HAADF-STEM images^37^.

In this letter, we report a TEM study of the crystal structure of quaternary Al-Cr-Fe-Si DQC. Both the structural characteristics of the long-distance tiling and the local clusters along the tenfold direction are investigated by using the HAADF-STEM images at an atomic resolution. Compared to the well-studied Al-Cu-Co, Al-Ni-Co, and Al-Mn-Pd DQCs, the quaternary Al-Cr-Fe-Si DQC shows different structural features, and may represent a new structural type of DQCs.

## Results and Discussion

**Figure 1:**
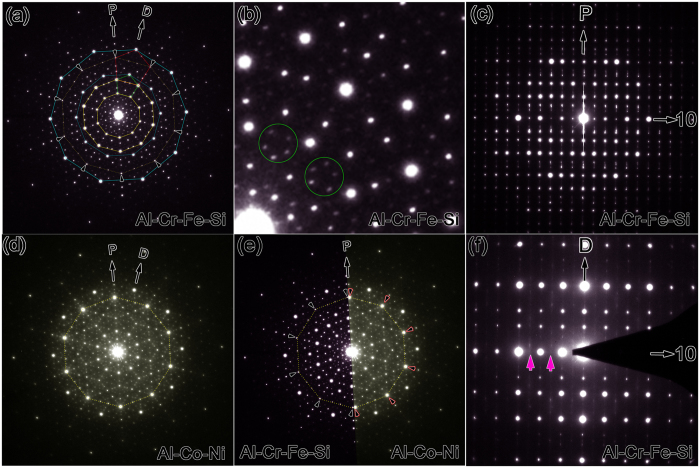
Selected-area EDPs of Al-Cr-Fe-Si DQC. (**a**) Along tenfold zone axis. The strong diffraction spots are linked by sold lines. Note that ten weak spots linked by dotted lines are strong in other Al-based DQCs. (**b**) An enlarged image of one portion of (**a**). (**c**) The EDP of one twofold zone axis. (**d**) The EDP of a Al-Co-Ni DQC along the tenfold projection. (**e**) Combined tenfold EDP from Al-Co-Ni and Al-Cr-Fe-Si alloys. (**f**) The EDP of another twofold zone axis differing 18° from the EDP in (**c**). Both the EDPs in (**c**,**f**) contain the periodic direction and perpendicular to the EDP in (**a**).

Figure 1a is the selected-area electron diffraction pattern (EDP) of a Al-Cr-Fe-Si DQC along the tenfold zone axis. The composition of the DQC is determined as Al_62_Cr_20_Fe_9_Si_9_ by energy dispersive X-ray spectroscopy (EDS). The strong diffraction spots show tenfold symmetry, as one of the evident features of DQCs. The Si element was previously found to facilitate the formation of quasicrystals^56^,^57^. Bancel *et al.* ascribed the main reason to the effects of Si on the gaps of electron band structure produced by pseudo-Brillouin zones^57^. We checked also the phases in the as-cast Al_70_Cr_20_Fe_10_ ingot, where the Si element of quaternary Al_60_Cr_20_Fe_10_Si_10_ alloy is substituted by Al. Besides some approximants of DQC, there is no DQC found in the as-cast Al_70_Cr_20_Fe_10_ sample, consistent with the Pavlyuchkov’s observations in the as-cast Al_72_Cr_16_Fe_17_ alloy^58^. Generally, the strong diffraction spots from the nearby concentric circles in the tenfold EDP of DQCs can form pentagons (a green one is shown in Fig. 1a as an example), and consequently a series of pentagons with the ratio of edge lengths close to τ = 1.618 were generated if one links also the strong diffraction spots in the tenfold EDPs of other Al-based DQCs^25^,^38^. However, the two of five spots of the red pentagon (~τ times of the green one), as marked by small arrowheads, is much weaker than the other three strong spots in our case. Similar phenomenon has also been observed from a thinner area with less dynamical diffraction effects of electrons. These weak spots locate in the direction along “P” (Fig. 1a) and form a decagon, as linked by dotted yellow lines. It is quite different from the other typical Al-based DQC systems such as Al-Co^14^, Al-Ni-Co^24^,^25^, Al-Pd-Mn^41^, Al-Cu-Co^19^, and Al-Ni-Ru^40^, because the diffraction spots in the corresponding positions in the latter systems are strong. We show here the corresponding EDP from an Al_74_Co_15_Ni_11_ alloy (nominal composition) in Fig. 1d as an example for comparison. The weak spots marked by arrows in Fig. 1a are replaced by strong spots (linked by one dotted yellow decagon) in the Al_74_Co_15_Ni_11_ alloy. The difference is more appreciable when we put the half of each EDP in Fig. 1a,d together, as shown in Fig. 1e. Therefore, the Al-Cr-Fe-Si DQC cannot be classified into any known Al-based DQCs so far, implying the presence of special structural features, which will be further studied by HAADF-STEM image at an atomic resolution. The abnormal strength of strong diffraction spots of two-dimensional quasicrystal has also been found in the quaternary Al-Co-Ni-Tb^59^, where the strong diffraction spots with fivefold rather than tenfold symmetry were observed along the direction corresponding to the tenfold axis of Al-Co-Ni DQCs. We also note that the weak diffraction spots inside the smallest green pentagon do not exactly locate the positions of a regular pentagon, as seen clearly in the circles in the enlarged image in Fig. 1b, which implies the structure of Al-Cr-Fe-Si DQC does not have a perfect reciprocal quasiperiodic lattice in comparing with the high-quality Al-Ni-Co^25^, and Al-Ni-Ru DQCs^40^. We ascribe the peak shifts of weak spots in Fig. 1a to the linear phason strains^60^,^61^,^62^,^63^, similar to that well known in other DQCs^41^,^64^. The translation period of the Al-Cr-Fe-Si DQC is determined as 1.23 nm from the two twofold EDPs in Fig. 1c,f, which are perpendicular to the tenfold projection in Fig. 1a. Note that the odd diffraction spots along the tenfold direction in Fig. 1f are extinction (two of them are indicated by purple arrowheads), implying a 10_5_ screw axis along this direction.

**Figure 2:**
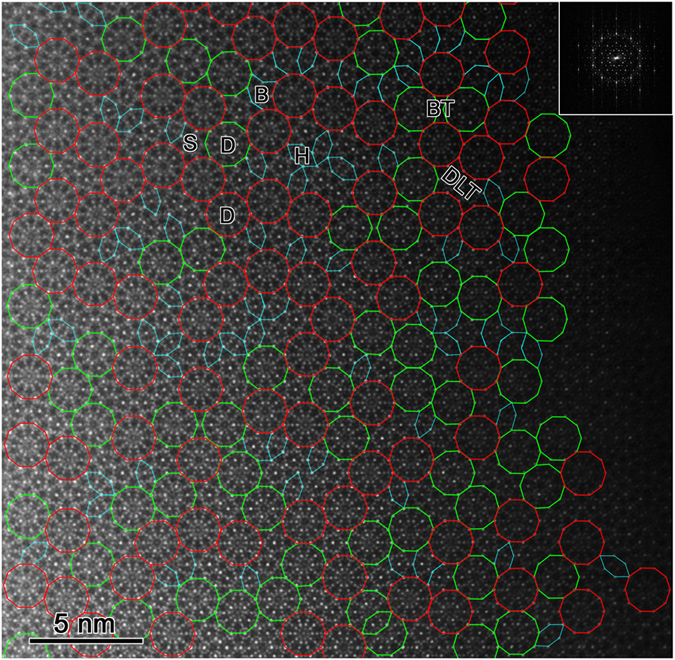
HAADF-STEM image along the tenfold axis of DQC. The structural blocks are depicted in color polygons. Among them, the D clusters are predominant and can be further classified into two types: with (in red) and without (in green) tenfold symmetry. The fast Fourier transform (FFT) of STEM image is inserted in the right-upper corner.

In order to reveal the structural characteristics of Al-Cr-Fe-Si DQC, we carried out HAADF-STEM studies at an atomic resolution by double aberration-corrected JEOL ARM 200F microscope. Figure 2 is a HAADF-STEM image along the tenfold axis of Al-Cr-Fe-Si DQC, with the structural blocks depicted in color polygons. The set of structural tiles contain decagon (D), star (S), boat (B), squashed hexagon (H), dumbbell-like tetradecagon (DLT), and bowtie (BT), as indicated in the image. The main structural blocks are the D clusters (in both red and green), where the red ones have the tenfold symmetry (Type I), and the green ones with the broken tenfold symmetry (Type II). Meanwhile, the quantity of Type I is more than that of the Type II. Structural differences of the Type II decagons will be discussed later in detail. Note that all the red and green decagons have the same orientation, different from the changeable orientations of the H and B tiles. Gummelt developed covering models to describe the structures of Al-based DQCs^26^,^65^. Base on the HAADF-STEM images, we suggest that the tiling model rather than Gummelt’s covering model is better to describe the structure of Al-Cr-Fe-Si DQC, where the former is also adopted for the structural description of Al-Ni-Rh DQC solved by X-ray diffraction^66^. As the primary structural block, the D clusters of Al-Cr-Fe-Si DQC connect to the neighboring ones by edge-sharing, without any covering. Furthermore, it is impossible to describe the structure of Al-Cr-Fe-Si DQC using only one kind of D structural blocks, unlike to the single D cluster as quasi-unit-cell to describe the Al-Ni-Co DQC^26^. The other tiles, e.g. S, B, etc., are indispensable to cover the whole plane of Al-Cr-Fe-Si DQC.

**Figure 3:**
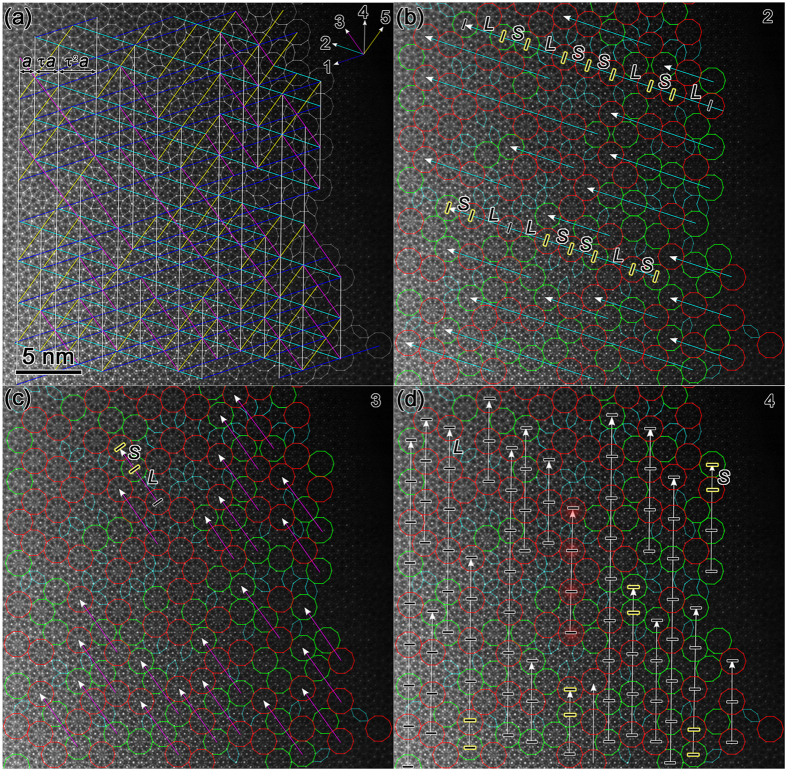
Alignment of D clusters along five directions differing by 36°. **(a)** The centers of at least three nearby D clusters in the same direction are connected in the same color lines. (**b**–**d**) Alignment of D clusters along directions 2 (**b**), 3 (**c**), and 4 (**d**), respectively, for comparison. The linked D clusters with the distances limited to the magnitude of the diameter of D cluster (marked by “*S*”) and the τ times of that (marked by “*L*”). Note that the periodic arrangement is found for D clusters along direction 4, but cannot be found along other directions.

The D clusters are aligned along five directions differing by 36° (as indicated by arrows in the upper-right corner in Fig. 3a), which is in agreement with the aligned clusters in the other Al-based alloys^23^,^27^. The connection of the centers of at least three D clusters results in a network constituted by five groups of parallel lines, where the distances of the parallel lines have ratios of ~τ and τ^2^, as indicated by one example in the upper-left corner in Fig. 3a. However, the line segments along the directions 3 (in purple), and 5 (in yellow) are shorter than those in the other directions generally, implying that these directions are not equivalent for the tiling of structural clusters.

In order to further demonstrate the variable arrangements of D clusters along different directions, we connected the centers of at least three nearby D clusters in the same direction to produce straight lines for comparison, but with the distances limited to the magnitude of the diameter of D cluster (marked by “*S*” in Fig. 3b–d) and the ~τ times of that (marked by “*L*”). Figure 3b shows the connections along the direction 2, with both long and short line segments. The combinations of “*S*” and “*L*” in the different long lines are changeable, e.g. the “*LL*” assembly is found in the lower line (as marked in Fig. 3b), but not found in the upper line (as marked). Differently, there are only short line segments along direction 3 (Fig. 3c), with the main combination of “*SL*”. However, the long line segments along direction 4 (Fig. 3d) are predominant. Differently, the distances of the nearby D clusters along this direction are almost the same (namely “*L*”), except five “S” (marked by black segments with yellow rim). Consequently, a local translational symmetry along one direction is found for some red D clusters, e.g. the four highlighted D clusters in the center in Fig. 3d. Therefore, the alignment of D clusters is tendentious in our case. Not only the quantity of aligned D clusters, but also the ratio of N_D_/N_L_ along direction 4 is higher than those along the other directions, where the N_D_ and N_L_ denote the number of aligned D clusters in each direction, and the number of lines with the same direction, respectively. These differences maybe relate with the irregular pentagon formed by weak spots in Fig. 1b.

**Figure 4:**
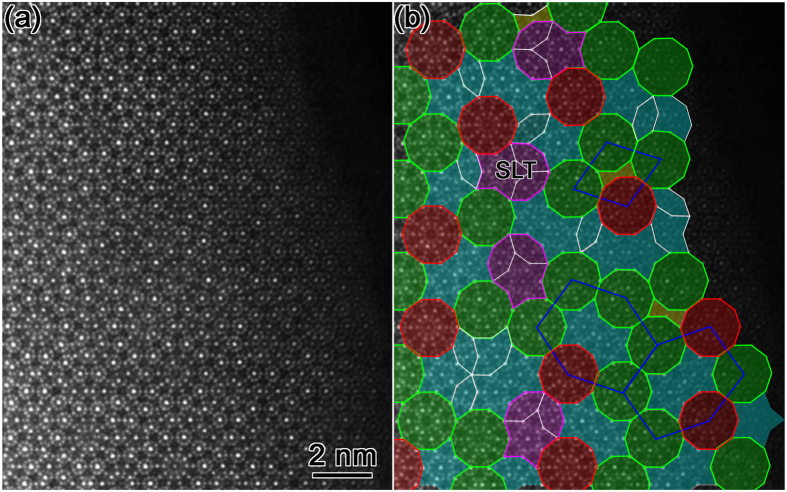
Enlarged HAADF-STEM image along tenfold projection. (**a**) The filtered image clearly shows the structural details of clusters. The brighter spots (TM atoms) are mainly found to be surrounded by ten weaker spots, forming a small decagon of 0.47 nm in diameter. (**b**) The structural units are depicted in colorful polygons. Again, the red and green D clusters represent tenfold and broken tenfold D clusters, respectively. Note that the D clusters in the vertexes of the fat rhombus and hexagon in blue are not equivalent.

For seeing the structural details, an enlarged HAADF-STEM image along the tenfold axis of DQC is shown in Fig. 4a. The structural tiles are superimposed in Fig. 4b, with again the Type I D clusters in red, while the Type II in green. The D cluster has a diameter of about 1.91 nm, a little less than the reported ~2.0 nm D clusters in the other systems such as Al-Ni-Co, Al-Cu-Co, and Al-Fe-Ni DQCs^7^. More difference in structures will be discussed in the following paragraph. Note that the two H and one S could further form a large cluster with a shield-like tiling (SLT), as highlighted in purple, which has been used to describe the structures of orthorhombic (3/2, 2/1) and (2/1, 3/2) approximants, with some merits compared to the structural subunits of H and S^67^. We see that the connection of the centers of D clusters can produce large rhombus, hexagon, and pentagon (could be found in Fig. 2) with an edge length of the diameter of D clusters (as outlined in blue lines in Fig. 4), which has been reported widely in other DQCs. In addition, the D clusters in the vertices of these shapes in our case are different, which were also noted in the other DQC alloys^68^.

**Figure 5:**
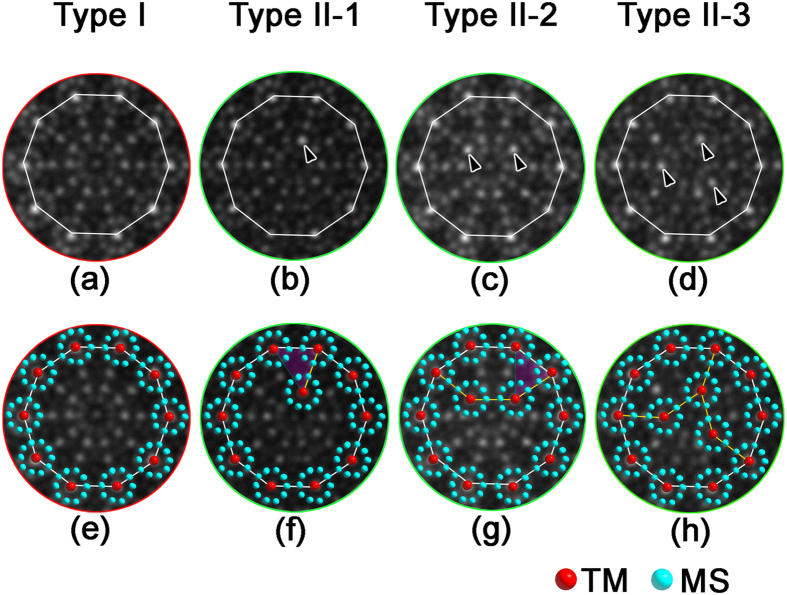
The D clusters with and without tenfold symmetry. Upper row: (**a**) Type I, with tenfold symmetry. (**b**–**d**) Type II, with broken tenfold symmetry. Lower row: the corresponding D clusters with the structural schematics of 0.47 nm clusters superimposed. The tenfold symmetry are gradually broken from left to right with the increase of the number of 0.47 nm clusters in the inner of large D clusters. The white decagon has a diameter of about 1.91 nm.

We now discuss the structural characteristics of the D clusters because they were widely observed in other Al-based DQCs^1^,^4^,^7^. Furthermore, the importance of the D clusters has simulated lots of studies and discussions^26^,^30^,^31^. The upper row in Fig. 5 is the enlarged D clusters of Types I, and II in the Al-Cr-Fe-Si system. The common feature of these clusters is that ten smaller clusters (with a diameter of ~0.47 nm) locate at the periphery of the 1.91 nm D cluster. The 0.47 nm clusters, with each one consisting of 10 weak dots (MSs) surrounding one brighter dot (TM) in the center, locate homogeneously the positions with a tenfold symmetry. The connection of the centers of these 0.47 nm clusters forms a decagon of 1.91 nm, as indicated by white lines. Figure 5a is one Type I D cluster. It has a tenfold symmetry, not only for the ten 0.47 nm clusters at the periphery, but also for the internal atoms. Although the D clusters with perfect tenfold symmetry observed in the other alloys such as in Al-Ni-Co^8^,^31^, and Al-Mn-Pd^8^, the structures of those reported clusters and those in the present case are different. For example, the ten 0.47 nm clusters do not exist in the D clusters in Al-Ni-Co alloys; the positions of TM atoms in the Al-Mn-Pd D clusters^8^ and ours are totally different. The TM atoms concentrate in the inner area of the ~2.0 nm D clusters in Al-Mn-Pd system (For more details, please refer to Fig. 21 in ref. 8), rather than in the centers of 0.47 nm clusters in our case.

The main types of the D clusters with broken tenfold symmetry (Type II) are shown in Fig. 5b,c. They are further classified into three subtypes: Type II-1, Type II-2, and Type II-3, respectively, because they are different in the inner structure. There is one more 0.47 nm cluster (with one arrow indicating the center in Fig. 5b) in the inner area of the Type II-1 D cluster, two more (as indicated by two arrows in Fig. 5c), and three more 0.47 nm clusters for Type II-2, and Type II-3, respectively, compared to the D cluster with perfect tenfold symmetry in Fig. 5a. The structural differences of these D clusters are clear at a glance when the structural model of the 0.47 nm cluster are superimposed onto the images, as seen in the lower row. Two 0.47 nm clusters in the periphery and one in the inner area form a triangle in Fig. 5f, as highlighted in purple. The position of the 0.47 nm cluster in the inner area could be well understood because this triangle is actually one part of the squashed H unit, as seen from the purple area in Fig. 5g (but the orientation is relatively rotated by 36° in the plane around the tenfold axis). One squashed H block is then generated when two more 0.47 nm clusters occurred in the inner area of the 1.91 nm D cluster, as seen in Fig. 5g. Consequently, the 1.91 nm D cluster could be decomposed into one squashed H and one concave decagon. Except the triangle area in Fig. 5f and the squashed H in Fig. 5g, the other parts of Type II-1, and Type II-2 are the same as the corresponding areas in the perfect D cluster in Fig. 5e. One more general 1.91 nm D cluster (Type II-3) is produced when three 0.47 nm clusters locate in the inner area of the 1.91 nm D cluster (Fig. 5h). It can be regarded as two squashed H and one B tiles, which are not only observed more often than the Type II-1, and Type II-2 D clusters, but also often found in the other DQCs.

We may see that the tenfold symmetry of the 1.91 nm D cluster is broken gradually from Fig. 5e–h, with increasing the number of 0.47 nm clusters in the inner area. The D clusters with broken tenfold symmetry in the Al_60_Cr_20_Fe_10_Si_10_ alloy are also different from those D clusters with broken tenfold symmetry in other Al-based alloys. For example, the broken tenfold symmetry in the Al-Co-Ni alloys is generally caused by the atoms around the centers of D clusters, where the arrangement of those atoms shows a fivefold symmetry or asymmetric distribution^36^. Note that we have discussed the main types of the D clusters here. We also notice that there are still a small amount of other D clusters, which deviate slightly in the contrast from the four kinds of D clusters mentioned above. Recently, several types of 2 nm D clusters with localized atomic disorder were found in the Al_58_Cu_26_Ir_16_ alloy^68^. However, the two types among the various D clusters of Al_58_Cu_26_Ir_16_ DQC, namely one with definite mirror symmetry and another with tenfold symmetry, are basic and the others could be considered as the liner combination of these two fundamental ones^68^. We note that the D clusters in Fig. 5 could be also classified into the two types where one with perfect tenfold symmetry (Type I), and another with mirror symmetry (Type II-1, -2, -3), similar to the classification of the two fundamental D clusters in Al_58_Cu_26_Ir_16_ DQC^68^.

In summary, a new type of DQC with the periodicity of 1.23 nm has been observed in a quaternary Al_60_Cr_20_Fe_10_Si_10_ alloy by TEM, in which the ten relatively weak diffraction spots in the EDP along the tenfold projection are found in the corresponding positions of strong diffraction spots in other Al-based DQCs. The structural characteristic at an atomic level is revealed by HAADF-STEM images. The structural blocks are composed of D, H, B, S, BT, and DLT, where the 1.91 nm D cluster is primary. The D clusters are various and can be mainly classified into two types: Type I, with perfect tenfold symmetry; Type II, where the tenfold symmetry is broken. Furthermore, the latter can be further classified into three types: Type II-1, Type II-2, and Type II-3, with one, two and three more 0.47 nm clusters, respectively, in the inner of D clusters. The neighboring D clusters share one edge, suggesting the structure is better to be described by tiling model rather than overlapping model. The D clusters are aligned along five directions differing by 36°, but the alignment is tendentious.

## Methods

An Al-Cr-Fe-Si alloy ingot of around 1 Kg with a nominal composition of Al_60_Cr_20_Fe_10_Si_10_ was first molten by melting high-purity Al (99.99 wt.%), and Fe (99.9 wt.%), Cr (99 wt.%), Si (99.3 wt.%) metals in an induction furnace under vacuum, and then the molten alloy in the furnace was poured into a copper mould to form an ingot. Powder samples were adopted for TEM observations. We firstly crushed small blocks from the ingot into powders. Then, alcohol was added into the powders to prepare suspension by following ultrasonic for 3 min. Finally, a drop of suspension was dripped onto a 3 mm copper grid covered by hollow carbon film for TEM observations. An FEI Tecnai F30 transmission electron microscope equipped with a Gatan 894 CCD camera is first used to search the interesting areas of DQCs and to take electron diffraction patterns (EDPs). The HAADF STEM images at an atomic resolution were done by a JEM-ARM200F microscope equipped with cold field emission gun, Cs-probe corrector and Cs-image corrector. The inner and outer acceptance semi-angle for HAADF-STEM imaging is 90 and 370 mrad, respectively. The HAADF-STEM images were processed by Fourier filter to decrease the noises of the original images. The Fourier process was carried out by Gatan Digital Micrograph software. A band pass mark was firstly added onto the fast Fourier transform (FFT) of the original STEM image to cover the diffraction spots, and then we applied filter option by keeping the masked area. The intensities of diffraction spots intensity are measured directly from the original diffraction patterns in “dm3” format by Gatan Digital Micrograph software. The composition was measured by the energy dispersive X-ray spectroscopy (EDS) using JEOL JED-2300 analysis station, with the detection area being 100 mm^2^ and the energy resolution 128 eV, equipped in the JEM-ARM200F microscope.

## Additional Information

**How to cite this article**: He, Z. *et al.* New type of Al-based decagonal quasicrystal in Al_60_Cr_20_Fe_10_Si_10_ alloy. *Sci. Rep.* **6**, 22337; doi: 10.1038/srep22337 (2016).
